# Prevalence of low bone mineral density in children and adolescents with celiac disease under treatment

**DOI:** 10.1590/S1516-31802009000500006

**Published:** 2010-02-03

**Authors:** Maria Eugênia Farias Almeida Motta, Maria Eduarda Nóbrega de Faria, Gisélia Alves Pontes da Silva

**Affiliations:** I MD, PhD. Adjunct professor, Division of Pediatric Gastroenterology, Universidade Federal de Pernambuco (UFPE), Recife, Pernambuco, Brazil.; II MD, MSc. Physician, Division of Pediatric Gastroenterology, Instituto Materno Infantil Professor Fernando Figueira (IMIP), Recife, Pernambuco, Brazil.; III MD, PhD. Associate professor, Division of Pediatric Gastroenterology, Universidade Federal de Pernambuco (UFPE), Recife, Pernambuco, Brazil.

**Keywords:** Celiac disease, Bone mineral density, Glutens, Diet therapy, Child, Therapeutics, Doença celíaca, Densidade mineral óssea, Glutens, Dietoterapia, Criança, Tratamento

## Abstract

**CONTEXT AND OBJECTIVE:**

Low bone mineral density may be a finding among children and adolescents with celiac disease, including those undergoing treatment with a gluten-free diet, but the data are contradictory. The aim of this study was to determine the frequency of bone mineral density abnormalities in patients on a gluten-free diet, considering age at diagnosis and duration of dietary treatment.

**DESIGN AND SETTING:**

Cross-sectional prevalence study at the Pediatric Gastroenterology Outpatient Clinic of Instituto Materno Infantil Professor Fernando Figueira.

**METHODS:**

Thirty-one patients over five years of age with celiac disease and on a gluten-free diet were enrolled. Bone mineral density (in g/cm^2^) was measured in the lumbar spine and whole body using bone densitometry and categorized using the criteria of the International Society for Clinical Densitometry, i.e. low bone mineral density for chronological age £ -2.0 Z-scores. Age at diagnosis and duration of dietary treatment were confirmed according to the date of starting the gluten-free diet.

**RESULTS:**

Low bone density for chronological age was present in 3/31 patients in the lumbar spine and 1/31 in the whole body (also with lumbar spine abnormality). At diagnosis, three patients with low bone mineral density for the chronological age were more than 7.6 years old. These patients had been on a gluten-free diet for six and seven months and 3.4 years.

**CONCLUSION:**

Pediatric patients with celiac disease on long-term treatment are at risk of low bone mineral density. Early diagnosis and long periods of gluten-free diet are directly implicated in bone density normalization.

## RESUMO

**CONTEXTO E OBJETIVO:**

Baixa densidade mineral óssea pode ser encontrada em crianças e adolescentes com doença celíaca, incluindo aqueles em tratamento com dieta sem glúten, mas dados são contraditórios. O objetivo deste estudo foi determinar a frequência de alteração da densidade mineral óssea nos pacientes em dieta sem glúten, conforme a idade ao diagnóstico e o tempo de tratamento dietético.

**TIPO DE ESTUDO E LOCAL:**

Foi realizado estudo transversal de prevalência no Ambulatório de Gastroenterologia Pediátrica do Instituto Materno Infantil Professor Fernando Figueira (IMIP).

**MÉTODOS:**

Trinta e um pacientes maiores de cinco anos com doença celíaca que estavam em dieta sem gluten foram avaliados. A densidade mineral óssea (em g/cm^2^) foi medida na coluna lombar e no corpo inteiro utilizando densitometria óssea, categorizando-a pelo critério da Sociedade Internacional para Densitometria Clínica (baixa densidade mineral óssea para a idade cronológica £ -2.0 escores Z). A idade ao diagnóstico e o tempo de tratamento foram confirmados pela data de início da dieta sem glúten.

**RESULTADOS:**

Baixa densidade mineral óssea para a idade cronológica foi detectada em 3/31 pacientes na coluna lombar e 1/31 no corpo inteiro (também apresentava alteração da coluna lombar). Ao diagnóstico, três pacientes com baixa densidade mineral óssea para a idade cronológica estavam com mais de 7,6 anos. Esses pacientes estavam em dieta sem glúten por seis e sete meses e 3,4 anos.

**CONCLUSÃO:**

Pacientes pediátricos com doença celíaca em tratamento a longo prazo são de risco para baixa densidade mineral óssea. Diagnóstico precoce e longo período de dieta sem glúten são diretamente implicados na normalização da densidade óssea.

## INTRODUCTION

Celiac disease is a chronic inflammatory enteropathy induced by exposure to gluten. It may be associated with decreased bone mineralization secondary to chronic inflammation of the intestinal mucosa. This causes poor absorption of calcium and vitamin D, which reduces serum calcium and stimulates the release of parathormone, thereby exacerbating bone reabsorption from the mobilization of bone calcium. It may also be associated with systemic inflammation, with increased concentrations of interleukin 1 and 6.^1^^,^^2^^,^^3^^,^^4^^,^^5^^,^^6^^,^^7^

Several studies have demonstrated that, independent of the clinical presentation, patients with untreated celiac disease present low bone mineral density (BMD).^1^^,^^2^^,^^3^^,^^5^^,^^7^^,^^8^ Osteopenia is a common public health problem in adults, but can be diagnosed and prevented in childhood. Considering that most of the bone mass is acquired during the first two decades of life, early diagnosis of celiac disease and adherence to a gluten-free diet are of fundamental importance for ensuring adequate bone metabolism in such cases.^9^^,^^10^ An early start to treatment for pediatric patients with celiac disease ensures significantly higher bone metabolism rates, since the treatment reverses the inflammatory process and prevents impairment of bone mass acquisition during the most important period for its acquisition.^3^^,^^5^^,^^11^ On the other hand, failure to adhere to a gluten-free diet reduces bone metabolism. There is controversy regarding the time required for a gluten-free diet to normalize bone mineralization. Some authors have detected this recovery over a short duration of adequate follow-up (up to one year), while others have stated that it can only be verified over a longer term.^1^^,^^3^^,^^7^^,^^11^^,^^12^^,^^13^

## OBJECTIVE

Since early diagnosis of celiac disease may avoid repercussions regarding bone metabolism, the present study was conducted with the aims of determining the frequencies of low BMD among children and adolescents with celiac disease under treatment and evaluating the presence of these abnormalities, considering the patient’s age at diagnosis, duration of treatment, failure to adhere to a gluten-free diet and indices of weight and height for age.

## METHODS

### Type of study

This was a cross-sectional prevalence study.

### Setting

The study was conducted at the Pediatric Gastroenterology Outpatient Clinic of a tertiary-level hospital, Instituto Materno Infantil Professor Fernando Figueira (IMIP), in Recife, Pernambuco, Brazil.

### Patients

The initial study population consisted of 37 patients aged 5 to 18 years who were undergoing treatment for celiac disease. Six patients aged less than five years were excluded because their bone densitometry could be evaluated with the pediatric software used in this study. The remaining 31 patients were evaluated for the presence of bone mineralization abnormalities. None of the patients was receiving calcium, vitamin supplements or drugs that could have altered their bone metabolism. This study was performed in accordance with the principles of the Declaration of Helsinki and it was approved by the Ethics Committee of IMIP. Informed consent was obtained from all of the patients’ parents.

### Procedures

The diagnosis of celiac disease had been made in accordance with the following criteria: clinical condition and first biopsy on the small intestine that were compatible with celiac disease, and/or clinical remission on withdrawal of gluten.^14^ The biopsy on the small intestine was performed by means of upper digestive tract endoscopy. The sample was analyzed and classified using the criteria of Marsh.^15^

The dual energy x-ray absorptiometry (DXA) method was used to perform bone densitometry on the lumbar spine and whole body except for the head, by using a DPX-L scanner (Lunar Corp, Madison, Wisconsin, United States). The patients were evaluated wearing trousers and a cotton shirt. All the measurements were made using the same apparatus, equipped with pediatric software (version 1.35), and were analyzed by a single experienced examiner. The bone mineral density (in g/cm^2^) of the lumbar vertebrae L2-L4 and whole skeleton was measured and categorized in accordance with the criteria of the International Society for Clinical Densitometry. Low bone density for chronological age was thus defined as less than or equal to -2.0 Z-scores.^16^

A questionnaire asking about signs and symptoms prior to the diagnosis and after starting the gluten-free diet was applied to the parents. One question asked whether there had been any failures to adhere to the diet over the preceding three months. Patients were deemed to be non-compliant with the gluten-free diet if they had had intake of gluten once a week or more frequently. A test for anti-human tissue transglutaminase (anti-tTG) antibody [ImmuLisa anti-human tissue transglutaminase antibody immunoglobulin A (IgA) Enzyme Linked ImmunoSorbent Assay (Elisa), IMMCO Diagnostics, United States] was performed on 23 patients in order to verify compliance with the gluten-free diet.

Weight and height measurements were made in accordance with the technique standardized by Gibson.^17^ Briefly, the patients were weighed while wearing underwear and t-shirt on a calibrated balance with a maximum capacity of 150 kg and sensitivity of 0.1 kg (Filizola, Brazil). Height was measured with the patients standing upright and keeping their eyes fixed on the horizon, using a stadiometer (professional model, Gofeka, Brazil), with an accuracy of 0.1 cm. The measurement of height was performed twice and the average measurement was recorded. The anthropometric indices of weight for age, height for age and body mass index (weight (kg)/height (m^2^)) were expressed as Z-scores, using the curves and graphs of the Centers for Disease Control and Prevention (CDC, Atlanta, United States) as the reference.^18^ The cutoff point for low weight and linear growth deficit was taken to be -2 Z-scores.^18^

In order to assess the role of the gluten-free diet in relation to BMD, the Mann-Whitney test (nonparametric data) was used to compare BMD (in g/cm^2^) in the lumbar spine and whole skeleton with categories of gluten-free diet periods (two, three and four years). Statistical significance was taken as P £ 0.05.

## RESULTS

The median age of the 31 patients (41.9% males) at the time of diagnosing celiac disease was 10.3 years (range: 5.2 to 18.7 years). This diagnosis was based on histopathological analysis in 25/31 cases (80.6%) and on clinical remission after starting a gluten-free diet in 6/31 cases (19.4%). At the time when the DXA was performed, the patients had been on a gluten-free diet for 0.6 to 10.1 years. The clinical manifestations at the time of diagnosis were diarrhea in 23/31 (74.2%) and abdominal distension and pain in 27/31 (87.1%), separately or in combination. At the time of the interview, all the patients were asymptomatic. Information about the patients’ age at diagnosis, beginning of the gluten-free diet, nutritional assessment, adherence to treatment and BMD are shown in Table 1.

Analysis of the lumbar spine using DXA showed low bone density in 3/31 patients (9.7%), while analysis of the whole skeleton identified low bone density in 1/31 patient (3.2%). This patient had both lumbar spine and whole body abnormalities. The patients with low bone density were diagnosed at the ages of 7.6, 11.6 and 14.3 years. With regard to the duration of the gluten-free diet, two patients presenting low bone density had been on a gluten-free-diet for six and seven months and one of them for 3.3 years. Failure to adhere to the gluten-free diet over the preceding three months was confirmed by three patients with low bone density. There were positive findings of anti-tTG antibodies in 47.1% (8/23) of the patients who underwent this test, and one of these presented low bone density.

The weight/age index was low in 6/31 patients (19.4%). The height/age index was low in 5/31 patients (16%), among whom one presented low bone density and had been on a gluten-free diet for seven months. This patient was positive for anti-tTG.

Analysis of the duration of the gluten-free diet showed that there was no statistical difference (P > 0.05) in the median BMD in the lumbar spine and whole skeleton for two and three years of gluten-free diet (data not shown). However, there was a trend towards significance with four years of gluten-free diet (Table 2).

**Table 1.** Clinical information and bone mineral density results for patients with celiac disease undergoing treatment

Patient	Age at diagnosis (years)	Duration of GFD^*^ (years)	Weight for age index (Z-score)	Height for age index (Z-score)	Body mass index (Z-score)	Adherence to diet	BMD^†^ lumbar spine (Z-score)	BMD^†^ whole skeleton (Z-score)
1	7.6 y	0.6 y	-4.74	-2.69	-4.69	no	-2.2	-2.6
2	9.8 y	1.10 y	-2.23	-2.29	-0.96	no	-0.6	-1.5
3	5.1 y	1.5 y	-3.89	-1.40	-5.64	yes	-0.8	-1.0
4	5.9 y	8.2 y	-1.22	-1.83	-0.21	yes	-1.2	-1.3
5	11.6 y	0.7 y	-1.80	-1.86	-0.87	no	-2.5	-3.1
6	2.9 y	8.9 y	0.18	-0.47	0.70	yes	1.0	1.1
7	0.4 y	5.9 y	2.77	0.65	3.20	yes	0.4	-0.6
8	1 y	7.3 y	-0.04	-0.79	0.51	yes	0.9	0.2
9	8.1 y	3.5 y	-2.07	-1.57	-1.47	yes	-1.5	-1.9
10	1.3 y	8.6 y	-1.47	-2.00	-0.29	no	0.5	-0.6
11	9.1 y	0.7 y	-2.29	-1.30	-2.30	no	0.6	-0.2
12	5.2 y	5.2 y	-1.69	-0.64	-2.14	yes	-0.9	-1.6
13	5.0 y	1.0 y	-1.31	-1.07	-0.81	yes	0.4	-0.4
14	9.2 y	1.4 y	-2.27	-2.33	-0.98	no	-0.6	-1.8
15	4.9 y	3.1 y	-1.79	-1.63	-1.05	yes	-0.3	-1.0
16	6.0 y	1.6 y	-0.69	-2.24	0.92	yes	-1.1	-1.8
17	1.7 y	8.10 y	-1.35	-0.19	-2.06	no	-1.4	-1.2
18	12.7 y	1.10 y	-1.56	-0.81	-1.48	yes	-1.0	-1.8
19	7.6 y	2.7 y	-1.66	-0.64	-2.10	yes	-1.3	-1.2
20	7.2 y	2.11 y	-0.59	-0.37	-0.36	yes	-1.3	-1.1
21	12.1 y	8.0 y	0.05	0.75	-0.26	no	-0.0	0.2
22	4.7 y	3.5 y	0.69	0.67	0.45	yes	-0.2	-0.5
23	2.6 y	10.1 y	-1.29	-0.22	-1.81	yes	-0.2	-0.1
24	1.7 y	7.10 y	-1.86	-1.26	-1.66	no	-0.3	0.3
25	3.1 y	7.1 y	-1.58	-0.56	-2.05	no	-0.5	-0.5
26	2.2 y	4.3 y	-0.54	-0.52	-0.28	yes	-0.4	-1.9
27	14.3 y	3.3 y	-1.13	0.29	-1.57	no	-3.9	-4.8
28	9.10 y	4.1 y	0.69	2.67	-0.53	yes	0.4	-0.3
29	10.2 y	0.11 y	-0.75	-0.24	-0.69	no	0.3	-0.0
30	4.8 y	8.3 y	-0.26	0.58	-0.81	yes	-0.5	-0.9
31	14.2 y	4.6 y	0.24	1.20	-0.41	no	-1.9	-0.8

^*^GFD = gluten-free diet; ^†^BMD = bone mineral density.

**Table 2.** Bone mineral density in lumbar spine and whole skeleton according to duration of gluten-free diet

Duration of gluten-free diet			
	≤ 4 years	> 4 years	P
Bone mineral density (g/cm^2^)			
Lumbar spine	0.625 (0.585-0.655)	0.721 (0.586-0.890)	0.11
Whole skeleton	0.808 (0.775-0.843)	0.868 (0.802-0.977)	0.07

## DISCUSSION

This study showed that some patients on a short-duration gluten-free diet for treating celiac disease can present low bone density for chronological age. These patients were diagnosed at older ages and had failed to adhere to the diet. One of them was found to be positive for anti-tTG and this patient’s weight-for-age and height-for-age indices were low. The sample in this study presented sufficient statistical power (95%): 31 patients were needed for this study, according to Statcalc (Epi-Info, version 6.04 b), with 25% of the patients presenting BMD lower than -2.5 Z-scores.^1^ Among our patients, 9.7% presented low BMD and thus this “worst result” was acceptable.

Diminished BMD is frequently found prior to diagnosing celiac disease.^1^^,^^2^^,^^3^^,^^4^^,^^5^^,^^8^^,^^19^^,^^20^ Just like in our study, other authors also found that when the gluten-free diet was started late, because of delays in diagnosing the disease, there was a relationship with lower BMD. Achieving normal bone mass was dependent on early diagnosis of the disease.^1^^,^^3^^,^^4^^,^^5^^,^^12^^,^^13^^,^^21^^,^^22^^,^^23^^,^^24^

There is currently a discussion in the literature about the duration of gluten-free diet that is needed to reverse the changes in bone metabolism relating to celiac disease. Recovery from bone abnormalities has been found to be correlated with the duration of the gluten-free diet.^1^^,^^2^^,^^3^^,^^11^^,^^13^^,^^19^^,^^25^ Increases in or stabilization of BMD have particularly been observed during the first year of the diet and are maintained over the long term (more than four years) in most patients.^1^^,^^2^^,^^3^^,^^4^^,^^5^^,^^11^^,^^13^^,^^19^^,^^20^^,^^24^ However, great variability in BMD during treatments for celiac disease have been described. This is related to the severity of atrophy of the villi at the time of diagnosis, the patient’s nutritional status and whether the diet has been followed strictly.^1^ Such variability may explain why some patients continue to present bone mineralization abnormalities, even over the long term, as detected in the present study.^1^^,^^21^^,^^26^ For this reason, it is still unclear whether low BMD due to celiac disease can be completely eliminated.^1^^,^^27^

The clinical and histological recovery is related to the extent of atrophy of the villi. Patients with severe histological lesions may achieve reductions in the severity of their lesions, to attain lower grades or even full recovery of the integrity of the mucosa after more than two years on a gluten-free diet. On the other hand, patients with mild lesions may achieve incomplete histological recovery, or may take years to recover, thus continuing to present persistent residual abnormalities.^25^^,^^28^^,^^29^ For this reason, it is questionable whether patients without symptoms after following a gluten-free diet are truly free from bone disease and have fully recovered their bone mass losses, since there is a tendency towards lower BMD indices in patients with almost total atrophy of the villi than in those with partial atrophy.^4^^,^^12^^,^^30^ A proportion of the patients who are asymptomatic after following a gluten-free diet continue to present reduced bone mineralization.^12^ Failure to adhere to the diet, celiac disease of greater severity due to late diagnosis, genetic factors and recurrent inflammatory stimuli on the intestinal mucosa from other causes may contribute towards delays in histological recovery and thus towards changes in bone metabolism.^28^ Most of the patients in the present study had severe histological lesions before starting on a gluten-free diet. It is possible to speculate that the patients who presented low bone density would be the ones who had not achieved full recovery from their histological lesions, thus continuing to present inflammation and consequently, BMD abnormalities. Although only eight cases were positive for anti-tTG, Tursi et al.^29^ did not observe a good correlation between anti-tTG and histological lesions. Thus, even the patients whose serological tests did not indicate any failure to adhere to the diet may have continued to have some degree of histological lesion.

It has been observed that patients who fail to adhere to the diet present low BMD, and this was also seen among our patients.^20^ In general, patients who follow a gluten-free diet without straying from it recover their BMD more rapidly, but simply complying with the diet does not ensure normalization, even after years.^2^^,^^5^^,^^8^^,^^19^^,^^20^^,^^21^^,^^25^^,^^30^^,^^31^^,^^32^^,^^33^ As is seen among chronic diseases in which dietary treatment is required, there is no guarantee that a gluten-free diet will be followed strictly over the long term, and failure to adhere to the diet was confirmed in 13 of our patients.^30^ Serological tests for celiac disease have been recommended as an indirect measurement for monitoring the compliance with the diet. Once the gluten has been eliminated and the symptoms have disappeared, its reintroduction causes no apparent symptoms.^34^ Anti-tTG antibodies form complexes with the gluten present in the diet and, after a short time on a gluten-free diet, the test for these antibodies become negative.^20^ This may elucidate why these antibodies were not detected in two of our patients with low BMD who said they had not been adhering to the diet, considering that such failures tend to be occasional rather than continuous and there is no single laboratory method to detect small dietary transgressions.^34^

The quantitative method most used for investigating bone mass in pediatrics is DXA. It is a method in which the measurements are based on two-dimensional projection of the three-dimensional bone structure, and this may induce measurement errors that are dependent on the size of the bone under evaluation.^11^^,^^21^^,^^35^ Thus, BMD abnormalities evaluated using DXA may only reflect the growth stage, such that the BMD of shorter individuals will be underestimated and the BMD of taller individuals will be overestimated.^11^^,^^19^^,^^25^ There needs to be caution in evaluating the presence of BMD abnormalities in patients with celiac disease who are undergoing treatment. Since low BMD is a chronic condition in which nutrient absorption and growth are abnormally low, it may be due to an inadequately treated underlying disease or it may just reflect inappropriate skeletal growth resulting from the length of time with the disease prior to starting on a gluten-free diet. Thus, catch-up growth may be impeded even when treatment is administered (an effect from chronic malnutrition).^11^^,^^12^^,^^13^^,^^36^ In our study, five patients presented abnormal weight-for-age indices, among whom one had low BMD. This may reflect nutrition that has been affected by the length of time with the disease, but it may also reflect the persistence of histological abnormalities due to inadequate adherence to the diet. On the other hand, since most of the patients of normal height did not present bone abnormalities, it is possible that in those with low BMD, this may have been the result of the celiac disease itself (late diagnosis, with insufficient length of treatment for histological recovery). The American Gastroenterology Association guidelines suggest that DXA scans are unnecessary in children with newly diagnosed uncomplicated celiac disease.^4^ Nonetheless, bone densitometry should be assessed in relation to each patient individually, and clinical evolution, histological findings and growth pattern should also be considered.

There were some limitations to our study. Firstly, not all of the patients were diagnosed by means of jejunal biopsies. However, the diagnostic criteria of the European Society of Paediatric Gastroenterology and Nutrition^14^ were followed, and these criteria are often used.^7^ Therefore, we believe that all of the patients included in the study had celiac disease. Secondly, the cross-sectional design may have led to difficulty in understanding the contributions of late treatment, good adherence to gluten-free diet and duration of treatment towards bone mass.

## CONCLUSION

This study detected that some patients with celiac disease who were on a gluten-free diet might present low bone density for chronological age, which could have resulted from older age at the time of diagnosis and short duration of gluten-free diet or inadequate compliance.
